# Associations between fruit and vegetable consumption and psychological distress: results from a population-based study

**DOI:** 10.1186/s12888-015-0597-4

**Published:** 2015-10-01

**Authors:** Aline Richard, Sabine Rohrmann, Caroline L. Vandeleur, Meichun Mohler-Kuo, Monika Eichholzer

**Affiliations:** Epidemiology, Biostatistics and Prevention Institute (EBPI), University of Zurich, Hirschengraben 84, CH-8001 Zurich, Switzerland; Centre for Research in Psychiatric Epidemiology and Psychopathology, Department of Psychiatry, University Hospital of Lausanne, Site de Cery, 1008 Prilly, Switzerland

## Abstract

**Background:**

Several studies observed associations of various aspects of diet with mental health, but little is known about the relationship between following the 5-a-day recommendation for fruit and vegetables consumption and mental health. Thus, we examined the associations of the Swiss daily recommended fruit and vegetable intake with psychological distress.

**Methods:**

Data from 20,220 individuals aged 15+ years from the 2012 Swiss Health Survey were analyzed. The recommended portions of fruit and vegetables per day were defined as 5-a-day (at least 2 portions of fruit and 3 of vegetables). The outcome was perceived psychological distress over the previous 4 weeks (measured by the 5-item mental health index [MHI-5]). High distress (MHI-5 score ≤ 52), moderate distress (MHI-5 > 52 and ≤ 72) and low distress (MHI-5 > 72 and ≤ 100) were differentiated and multinomial logistic regression analyses adjusted for known confounding factors were performed.

**Results:**

The 5-a-day recommendation was met by 11.6 % of the participants with low distress, 9.3 % of those with moderate distress, and 6.2 % of those with high distress. Consumers fulfilling the 5-a-day recommendation had lower odds of being highly or moderately distressed than individuals consuming less fruit and vegetables (moderate vs. low distress: OR = 0.82, 95 % confidence interval [CI] 0.69-0.97; high vs. low distress: OR = 0.55, 95 % CI 0.41-0.75).

**Conclusions:**

Daily intake of 5 servings of fruit and vegetable was associated with lower psychological distress. Longitudinal studies are needed to further determine the causal nature of this relationship.

## Background

Mental diseases are a leading cause of the global burden of disease and highly contribute to life-years lost [1, 2]. According to the World Health Organization (WHO), worldwide 25 % of individuals develop one or more mental or behavioral disorders during their lifetime [3].

Acute and chronic stress are considered to be potential risk factors for mental disorders, including depression and anxiety, depending on the individual’s stress sensitivity [4, 5]. There are several definitions of stress and/or psychological distress, which share the point of view that it is expressed by emotional suffering [6].

There is growing evidence that modifiable lifestyle factors, particularly diet, have a beneficial effect on the occurrence and recurrence of mental diseases, such as depression [7, 8]. Nevertheless, associations between diet and mental health are evaluated by a variety of different aspects, such as focusing on single dietary components, dietary patterns or on single nutrients [9–13].

In fruit and vegetables, there are a large number of bioactive compounds that could be responsible for an effect on mental health [14]. Nevertheless, only a few studies explicitly examined the association of fruit and vegetable intake with mental health, but these studies observed inverse associations [15, 16].

The 5-a-day recommendation is one of the best-known dietary campaigns to date, which takes fruit and vegetable intake into account and was implemented in the 1990s by the National Cancer Institute in the U.S. In the following years, many western countries engaged in similar campaigns (e.g. Germany, Great Britain, France). In Switzerland, the 5-a-day campaign started in 2001 and is defined by consuming at least 3 portions of vegetables and 2 portions of fruit daily. Other countries have slightly different definitions of 5-a-day (e.g. 5 daily portions without defining whether fruit or vegetables in France). Definitions rely on the WHO Global Strategy on Diet, Physical Activity and Health that recommends “a minimum of 400 g of fruit and vegetables per day (excluding potatoes and other starchy tubers)” [17]. To our knowledge, associations between 5-a-day recommendations and mental health have not been evaluated yet. Thus, given the paucity of research on the topic, it is worthwhile to evaluate whether the 5-a-day recommendation has a positive effect on mental health. Our aim was to examine the association between the adherence to the 5-a-day recommendation and psychological distress in the Swiss population.

## Methods

### Study population and data

Data were obtained from the Swiss Health Survey (SHS) conducted in 2012/2013 by the Swiss Federal Bureau of Statistics (SFSO). All data used for this study were collected by telephone interview. The data collection and data storage for the Swiss Health Survey does not require formal approval by an ethical committee. This data collection is specifically permitted under Swiss law (SR 431.012.1 and SR 431.112.1). Individuals invited to participate received a brief description of the study and could decline to participate or withdraw at any time. Participants’ responses were treated confidentially and aggregated anonymous responses were utilized for analyses presented herein.

The SHS is a cross-sectional, population-based nationwide survey on health status, several lifestyle and demographic factors, and healthcare use and has been carried out every five years since 1992. Using a stratified random sampling technique based on registries of inhabitants, individuals aged 15 years or older and living in a private household were recruited. A total number of 21,597 individuals participated, derived from an initial sample of 41,008 individuals (participation rate 54 %). A computer-assisted telephone interview (CATI) was performed and in a further step, a written questionnaire was provided (paper or online) upon approval from the participants (*n* = 18,357) [18]. This multistage probability sample can be considered as representative of the Swiss population.

Information on the mental health index (MHI-5) was available for 20,652 individuals. Individuals with missing information on fruit and vegetable consumption were excluded from analyses (*n* = 90). In a further step, we excluded individuals with missing information on covariates, such as age or education level etc. (*n* = 342), resulting in a final sample of 20,220 participants.

### Outcome measure

The outcome of interest was psychological distress measured by the 5-item mental health index [MHI-5] [19]. The five-item Mental Health Inventory (MHI-5) assessed the extent of perceived psychological distress during the previous 4 weeks. The MHI-5 is a valid tool to measure mental health in the general population and the five items assess how often over the past month individuals felt nervous, felt so down that nothing could cheer them up, felt calm and peaceful, felt down and blue, or felt happy [19–21]. Answers were categorized according to a 5-point Likert scale ranging from “always” to “never”. The MHI-5 has shown good sensitivity and specificity for detecting DSM-IV Axis-I disorders in the general population [22].

The SFSO provided the linearly transformed scale for the MHI-5, which ranges from 0 to 100 [23]. Studies have shown that scores below 53 indicate clinically relevant distress symptomatology (high distress), scores between 53 and 72 may indicate a higher probability of psychiatric symptoms but less than those for high distress (moderate distress), and scores above 72 are considered to represent good mental health status (low distress) [24–26]. The use of these 3 categories has also been recommended by the SFSO [23].

### Exposure measurements

Definitions of fruit and vegetable consumption were based upon food frequency questionnaires. For both fruit and vegetables, two questions were asked. The first question was related to frequency: “On how many days a week do you usually eat fruit or drink fruit juices?” or “On how many days a week do you usually eat vegetables or salad or drink vegetable juices (potatoes do not count)?”. Answers were coded as “less”, “rarely”, “1”, “2”, … to “7” times a week. The second question was related to the number of portions consumed: “And how many portions of fruit or fruit juices do you consume on average per day? One portion would be as big as a handful (i.e. 1 apple, 1 pear). For juice it is about 2 dl.”and “And how many portions of vegetables, salad or vegetable juices do you consume daily on average? One portion would be as big as a handful (or about 1 tomato, 1 big carrot). For juice it is about 2 dl”. The second question was only asked if the first question was positive for a frequency of at least “5 times a week” and answers were coded into “less than 1 portion”, and “1”, “2”, “3”, “4” and “5 portions or more”.

Based on the recommendations of the Swiss Nutrition Society [27] we defined adherence to the recommended amount of fruit and vegetable consumption as at least two and three portions per day, respectively. Adhering to both the recommended fruit and vegetable consumption was defined as compliance with the 5-a-day recommendation.

### Covariates

Sociodemographic variables and health behaviors that could influence the associations between fruit and vegetable consumption and psychological distress were examined as confounders and were included in the multivariable analyses. For body mass index (BMI), we differentiated between underweight, normal weight, overweight and obesity (<18.5 kg/m^2^, 18.5 - <25 kg/m^2^, 25 - <30, ≥30 kg/m^2^, respectively) [28]. For individuals younger than 18 years, the tables of TJ Cole, MC Bellizzi, KM Flegal and WH Dietz [29] were used to define the four BMI categories. We additionally included age categories (15–24, 25–34, 35–44, 45–54, 55–64, 65–74, 74+ years), gender, nationality (Swiss vs. foreigner), marital status (single, married, divorced/separated/widowed), educational level (low: compulsory education or less, middle: secondary education, high: tertiary education), smoking status (never, former, current), alcohol consumption (≤20 g ethanol per day for women, ≤40 g for men vs. >20 g, > 40 g, respectively) [30], physical activity (≥150 min. per week vs. less) [31], and chronic diseases (hypertension or diabetes; yes vs. no). Other types of food consumption were further dichotomized into unfavorable vs. recommended according to the Swiss Nutrition Society depending on the information about consumption frequencies. We included meat (>4 days per week vs. less), fish (never or less than one day per week vs. more) and dairy products (<2 portions per day vs. more) [27].

### Statistical analyses

All statistical analyses were conducted using STATA software version 13.1 (College Station, Texas). Sociodemographic and health-related characteristics were computed using contingency tables stratified by level of psychological distress. We conducted maximum-likelihood multinomial (polytomous) logistic regression analyses to determine associations between fruit and vegetable intake and levels of psychological distress (low vs. moderate and high, respectively). Results for multinomial regression analyses were computed in terms of relative risk ratios, but we use the term odds ratio (OR) to enhance comprehensibility. Four Models were conducted successively: 1) unadjusted, 2) adjusted for age and sex, 3) adjusted for demographic and health-related factors, and 4) adjusted for all the covariates in model 3 plus meat, fish and dairy product consumption. In order to draw valid conclusions regarding the Swiss population based on our sample, the SFSO made a comparison with the permanent 2012 Swiss population. All analyses were weighted by using the population-based weights of the telephone interviews provided by the Swiss Federal Office of Statistics. The weights are based on the 2012 Swiss population with respect to sex, age, geographic region and nationality (Swiss/non-Swiss); any differences caused by stratification or non-participation were mathematically corrected. Additionally, a sensitivity analysis was performed to compare non-included individuals who had information on their level of distress with those who were included in the present analysis. We also conducted an analysis that included interaction terms to assess the associations of sex and 5-a-day adherence with the psychological distress outcomes to determine whether the results differed between men and women.

## Results

Table 1 shows socio-demographic and health-related characteristics stratified by levels of psychological distress into low (82 %), moderate (13.4 %), and high (4.6 %). Among individuals with high distress levels, a higher percentage of individuals reported to be foreigners than individuals with moderate or low distress levels. A higher percentage of participants with high distress levels had a high level of education compared to individuals reporting low and moderate distress levels. Obesity tended to be reported more frequently by individuals with high distress levels than by individuals with low distress levels. A sensitivity analysis revealed similar distributions of sociodemographic characteristics stratified by psychological distress levels in individuals not included in this analysis (having missing information on fruit and vegetable consumption or confounders; *n* = 432; data not shown).

**Table 1:** Characteristics^a^ of the participants stratified by psychological distress levels^b^ from the Swiss Health Survey [50]

Distress level		low	moderate	high
n, unweighted		16,552	2721	947
Age (years), mean (SD)		47.0 (0.18)	46.8 (0.50)	46.6 (0.67)
		*%*	*%*	*%*
Total		82.0	13.4	4.6
Age (years)	15–24	13.8	13.2	8.9
	25–34	15.6	17.1	16.4
	35–44	16.7	17.1	20.1
	45–54	19.2	18.4	24.4
	55–64	13.8	14.7	15.2
	65–74	11.9	9.1	9.1
	75+	9.0	10.3	5.9
Nationality	Swiss	80.3	73.4	68.2
	Foreigner	19.7	26.6	31.8
Marital status	Single	32.6	32.3	29.6
	Married	51.6	48.2	46.3
	Divorced/separated/widowed	15.8	19.5	24.1
Educational level	Low	14.7	21.0	27.7
	Middle	54.2	54.8	54.8
	High	31.2	24.2	17.4
BMI kg/m^2^	<18.5	3.4	4.0	5.3
	≥18.5 - < 25.0	56.3	54.9	50.4
	≥25.0 - < 30.0	30.7	30.6	29.8
	≥30	9.6	10.5	14.4
Smoking history	Never	51.2	45.4	39.6
	Former smoker	22.0	21.5	17.1
	Current smoker	26.8	33.1	43.4
Moderate physical activity	<150 min. per week	24.5	36.0	45.1
	≥150 min. per week	75.5	64.0	54.9
Alcohol consumption^c^	Hazardous chronic consumption	4.7	6.0	4.2
	Lower consumption or none	95.3	94.0	95.8
Chronic diseases^d^	No	73.1	69.2	67.3
	Yes	26.9	30.8	32.7
Pay attention to diet	Yes	68.7	68.6	71.4
	No	31.3	31.4	28.6
Milk and/or dairy products	<3 portions daily	90.3	91.8	90.0
	≥3 portions daily	9.7	8.2	10.0
Fish	Never/less than one day per week	34.9	34.1	35.1
	More	65.1	65.9	64.9
Meat	≥4 times weekly	39.1	34.1	33.2
	<4 times weekly	60.9	65.9	66.8

^a^Values are self-reported and weighted except n

^b^Low distress (MHI-5 > 72 & ≤ 100), moderate distress (MHI-5 > 52 and ≤ 72), and high distress (MHI-5 ≤ 52)

^c^≤ 20 g ethanol daily for women, and ≤ 40 g ethanol daily for men

^d^Hypertension and diabetes

As we did not observe any statistically significant effect modification by sex (all interaction terms > 0.05; data not shown), only the results of the overall sample are reported.

**Fig. 1:**
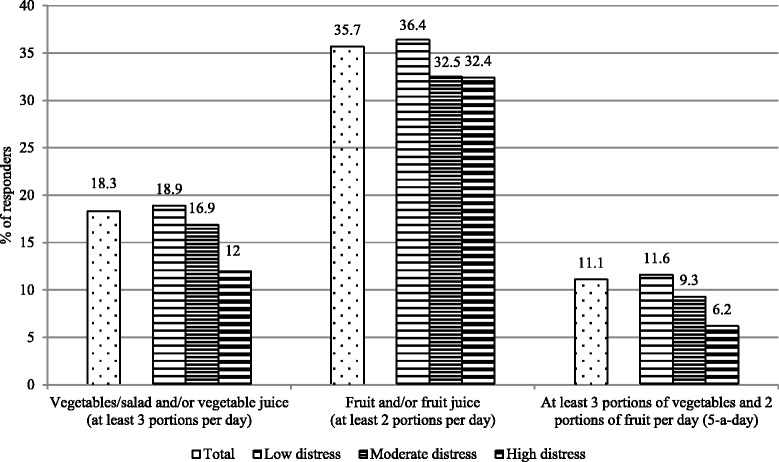
Consumption of fruit and vegetables overall and stratified by psychological distress level of the Swiss Health Survey [50]

Figure 1 shows that the consumption of at least 3 portions of vegetables per day was reported by 18.3 % and of at least 2 portions of fruit per day was reported by 35.7 % of all individuals. The 5-a-day recommendation was fulfilled by 11.1 % of all individuals, and when stratified by distress levels, was reported by a higher percentage of individuals with low distress compared to individuals with moderate or high distress levels.

Table 2 reveals that participants adhering to the recommended amount of vegetable consumption had a lower odds of reporting high distress levels compared to those who were non-adherent (odds ratio [OR] 0.64; 95 % confidence interval [CI] 0.49–0.82, multivariable adjusted plus diet; Table 2). For moderate distress levels, these results were no longer statistically significant after multivariable adjustment.

**Table 2:** Associations between fruit and vegetable intake and psychological distress in the Swiss population (Swiss Health Survey [50]); multinomial logistic regression^a^

	*Total (n = 20,220)*				
Distress level	low	moderate		high	
		OR	95 % CI	OR	95 % CI
Vegetables/salad and/or vegetable juice (at least 3 portions per day)					
unadjusted model	1	0.87	[0.76,1.00]	0.58	[0.46,0.74]
age and sex adjusted model	1	0.81	[0.70,0.93]	0.47	[0.37,0.61]
multivariable adjusted model^b^	1	0.93	[0.80,1.07]	0.64	[0.50,0.83]
multivariable adjusted model plus diet^c^	1	0.93	[0.81,1.07]	0.64	[0.49,0.82]
Fruit and/or fruit juice (at least two portions per day)					
unadjusted model	1	0.84	[0.76,0.94]	0.84	[0.70,1.00]
age and sex adjusted model	1	0.80	[0.71,0.89]	0.75	[0.62,0.90]
multivariable adjusted model^b^	1	0.84	[0.75,0.94]	0.85	[0.70,1.03]
multivariable adjusted model plus diet^c^	1	0.83	[0.74,0.93]	0.84	[0.69,1.02]
At least 3 portions of vegetables and 2 portions of fruit per day (5-a-day)					
unadjusted model	1	0.78	[0.66,0.91]	0.50	[0.38,0.67]
age and sex adjusted model	1	0.71	[0.60,0.84]	0.41	[0.30,0.54]
multivariable adjusted model^b^	1	0.82	[0.69,0.97]	0.56	[0.41,0.76]
multivariable adjusted model plus diet^c^	1	0.82	[0.69,0.97]	0.55	[0.41,0.75]

^a^Weighted

^b^Adjusted for age, sex, nationality, smoking status, alcohol consumption, body mass index, physical activity, chronic diseases, education

^c^Adjusted for age, sex, nationality, smoking status, alcohol consumption, body mass index, physical activity, chronic diseases, education, meat consumption, fish consumption, dairy products consumption

We did not observe a statistically significant association between adherence to fruit consumption and the odds of high distress levels (OR 0.84; 95 % CI 0.69–1.02), but participants who consumed the recommended amount of fruit were statistically significant less likely than those who were non-adherent to report moderate distress levels (OR 0.83; 95 % CI 0.74–0.9, multivariable adjustment plus diet; Table 2).

Adhering to the 5-a-day recommendation was statistically significantly associated with high and moderate distress levels compared to non-adherence throughout all the models. In the multivariable adjusted model plus diet, e.g., adhering to the 5-a-day recommendation was statistically significantly associated with lower odds of high distress (OR 0.55; 95 % CI 0.41–0.75) and moderate distress levels (OR 0.82; 95 % CI 0.69–0.97), respectively.

## Discussion

In this large population-based Swiss survey we observed significant inverse associations between fruit and vegetable consumption and distress levels. Individuals keeping to the 5-a-day recommendation had a lower likelihood to report moderate or high distress levels than individuals not adhering to the 5-a-day recommendation. To our knowledge, this is the first time associations of this type are reported in Switzerland. Our results are in line with the few existing Western country studies on the effect of fruit and vegetable intake on mental health. In a cross-sectional random sample of nearly 1000 primary care patients in the US, Rohrer et al. [32] observed that a higher self-reported quantity of fruit and vegetables per day was associated with lower mental distress. McMartin et al. [16] also observed an association between fruit and vegetable consumption and mental health using five waves of a national cross-sectional Canadian survey. A recent cohort study on women’s health from Australia found an inverse effect of fruit and vegetable consumption on incident depression after a 6-year follow-up [33]. Another 12-year follow-up study in a cohort of generally healthy Australian men and women observed that adherence to a Mediterranean-style diet was associated with less psychological distress at follow-up [34].

A recent review of observational studies including cohort, case–control and cross-sectional studies came to the conclusion that “healthy” and Mediterranean dietary patterns seem to lower the likelihood of depression [11, 35]. An important part of a Mediterranean diet is a high intake of fruit and vegetables [36]. In a number of studies, diet quality was evaluated in relation to depression [37–39]. However, dietary patterns were assessed quite heterogeneously. Additionally, in studies primarily looking at single dietary components, such as fruit and vegetables, analyses were often not controlled for other dietary or lifestyle factors [15, 16, 40].

In our study, we performed two sets of multivariable analyses, i.e. one including age, sex, education, nationality, smoking status, physical activity and chronic diseases and an additional one further including additional dietary factors, i.e., consumption of fish, meat, and dairy products, as potential confounders. The results remained similar, thus, strengthening the evidence that healthy fruit and vegetable consumption might be responsible for the observed inverse association with mental distress.

Fruit and vegetables are rich in antioxidants such as vitamin C, vitamin E, carotenoids, phenolic compounds etc. [14]. Antioxidants have two main effects. First, they reduce oxidative stress [41]. Oxidative stress, in turn, has consistently been shown to be increased in chronic stress and depression [42]. Second, antioxidants in diet can decrease inflammation such as cytokine production [41, 43]. There is some evidence that cytokine production is elevated in stress and depression, but the higher concentrations could also be the consequence of additional diseases or the consumption of drugs [44, 45]. Nevertheless, studies examining the association of antioxidants with depression are still rare but seem to support an inverse association. For example, the InCHIANTI cohort study found that low plasma concentrations of carotenoids were significantly associated with incident depression in older individuals over a 6-year follow-up [44].

Folate is a further substance in fruit and vegetables that has been shown to be linked to depression. A meta-analysis of observational studies showed significant inverse associations of folate status with depression [46]. The included studies were mostly cross-sectional, but the result was also supported by one cohort study [46]. The latter study hypothesized that folate increases methylation processes and the regulation of neurotransmitters, such as serotonin which, in turn, is associated with a lower risk of depression.

### Strengths and limitations

Our analyses were based on the MHI-5, whereas research on diet and depression is more common than on diet and mental distress in general. However, the MHI-5, which assesses distress over the previous 4 weeks, was compared to clinical interviews as the gold standard and has been shown to be a valid tool to detect depression in the general population as well as in psychiatric surveys [19, 22, 26]. Furthermore, our results may have practical implications not only for individuals with high psychological distress, who, according to the MHI-5 would receive a diagnosis of mental disorders, but also for individuals with moderate distress, who are considered vulnerable to the development of mental disorders, especially depression and anxiety [4].

A further strength of our study is the multivariable adjustment for potential confounders, and in particular for other dietary factors. In most fruit and vegetables, omega-3 fatty acid concentration is negligible, but there is a large amount of omega-3 fatty acids in some types of fish, which were mostly associated with depression in cross-sectional and prospective epidemiological studies as well as in randomized controlled trials [8, 13]. We partly accounted for this by adjusting our analysis for fish consumption and our results remained unchanged. Nevertheless, residual confounding, and confounding due to other dietary factors such as energy intake, which were not assessed in the SHS, cannot be excluded. Additionally, the questions about diet were not validated. A further limitation is that the direction of the association cannot be derived, due to our cross-sectional design. It might be possible that the presence of mental distress could result in dietary changes [47]. This is for example supported by the possibility of “loss of appetite” in depressive episodes as defined in the international statistical classification of diseases and related health problems (ICD-10) [48] which may affect eating patterns. However, the results of two prospective studies, have strengthened the evidence that healthy diet [49] and fruit consumption in particular [33] have an impact on subsequent mental health.

## Conclusions

Keeping to the 5-a-day recommendation was associated with lower psychological distress. Thus, strengthening efforts to comply with this dietary recommendation would be an effective and cost-effective means to lowering psychological distress. Nevertheless, these findings warrant confirmation in prospective studies, specifically to establish the temporal sequence of this association.

### Ethical standards

Legal basis: Ordenance of the Conduct of Federal Statistical Surveys of June 30, 1993
